# Prognostic values of four Notch receptor mRNA expression in gastric cancer

**DOI:** 10.1038/srep28044

**Published:** 2016-07-01

**Authors:** Xiaoyu Wu, Wentao Liu, Ding Tang, Haijuan Xiao, Zhenfeng Wu, Che Chen, Xuequan Yao, Fukun Liu, Gang Li

**Affiliations:** 1 Department of Surgical Oncology, Affiliated Hospital of Nanjing University of Traditional Chinese Medicine, Nanjing 210029, P.R. China; 2 Department of Surgery, Ruijin Hospital, Shanghai Jiaotong University, Shanghai Institute of Digestive Surgery, Shanghai 200025, P.R. China; 3 Biomedical Engineering, School of Engineering, Sun Yat-Sen University, Guangzhou 510120, P.R. China; 4 Department of Oncology, Hospital Affliated to Shaanxi University of Chinese Medicine, Xianyang 712000, P.R. China; 5 Department of General Surgery, Jiangsu Cancer Hospital, The Affiliated Cancer Hospital of Nanjing Medical University, Nanjing 210009, P.R. China

## Abstract

Notch ligands and receptors are frequently deregulated in several human malignancies including gastric cancer. The activation of Notch signaling has been reported to contribute to gastric carcinogenesis and progression. However, the prognostic roles of individual Notch receptors in gastric cancer patients remain elusive. In the current study, we accessed the prognostic roles of four Notch receptors, Notch 1–4, in gastric cancer patients through “The Kaplan-Meier plotter” (KM plotter) database, in which updated gene expression data and survival information include a total of 876 gastric cancer patients. All four Notch receptors’ high mRNA expression was found to be correlated to worsen overall survival (OS) for all gastric cancer patients followed for 20 years. We further accessed the prognostic roles of individual Notch receptors in different clinicopathological features using Lauren classification, pathological grades, clinical grades, HER2 status and different choices of treatments of gastric cancer patients. These results indicate that there are critical prognostic values of the four Notch receptors in gastric cancer. This information will be useful for better understanding of the heterogeneity and complexity in the molecular biology of gastric cancer and to develop tools to more accurately predict their prognosis.

Gastric cancer is the second most common cause of cancer-related death, and 800,000 cancer-related deaths are caused by gastric cancer each year globally^1^ Despite the advances in early detection, radical cure operation, and multimodal therapeutic modalities, at diagnosis, gastric cancer remains difficult to cure and prognosis remains poor with a median overall survival of 12 months for advanced disease in Western countries^2^,^3^. About 40–60% patients with gastric cancer received radical operation will often have postoperative recurrence and metastasis^4^. Therefore, in order to improve the clinical outcome of gastric cancer patients, the identification of the molecular mechanism during the incidence and progression of gastric cancer, as well as identification of prognostic biomarkers and drug targets are still needed and will help to select patients with higher chances of gastric cancer recurrence and provide better prognosis and individualized treatments.

The Notch signaling pathway is one of key pathways constituting the stem cell signaling network and is a highly conserved system that regulates cell-fate decisions and the maintenance of stem cells^5^,^6^. DLL1, DLL3, DLL4, JAG1 and JAG2, typical Notch ligands, and four Notch receptors (Notch 1–4) are frequently deregulated in several human malignancies and have been found in breast, colon, cervical, head and neck, lung, pancreatic, prostate cancer, ovarian cancer, renal carcinoma, acute myeloid, Hodgkin and Large-cell lymphomas, as well as gastric cancer^7^,^8^,^9^,^10^,^11^,^12^. A number of studies have shown that the activation of Notch signaling plays a critical role in gastric cancer initiation, progression and cross-talks with other signaling pathways, contributing to the apoptosis inhibition, development, angiogenesis, metastasis and chemoresistance of gastric cancer^7^,^13^,^14^,^15^,^16^,^17^,^18^. However, the prognostic roles of individual Notch receptors, especially at the mRNA level in gastric cancer patients remains elusive. In the current study, we accessed the prognostic roles of four Notch receptors in human gastric cancer patients by the Kaplan-Meier plotter (KM plotter).

KM plotter generated data from Gene Expression Omnibus (GEO-www.ncbi.nlm.nih.gov/geo/) database. This database includes gene expression data and survival information from a total of 876 gastric cancer patients. KM plotter can be utilized for the analysis of individual genes with clinical results to relapse-free survival and total survival of the patients. So far, a number of genes have been identified and/or validated by KM plotter in lung cancer^19^,^20^,^21^,^22^, breast cancer^22^,^23^,^24^,^25^,^26^,^27^,^28^,^29^,^30^,^31^,^32^, and ovarian cancer^22^,^33^,^34^. In this study, we used KM plotter database and accessed the prognostic roles of individual Notch receptor mRNA expression in human gastric cancer patients.

## Material and Methods

An online database^35^ (http://kmplot.com/analysis/) was used to determine the relevance of individual Notch receptor mRNA expression to the overall survival (OS). OS is the length of time from either the date of diagnosis or the start of treatment for a cancer patient, that patients diagnosed with the cancer are still alive. In a clinical trial, measuring the OS is one of important ways to see how well a new treatment works. Currently, they established breast cancer^35^, lung cancer^19^, ovarian cancer^36^ and gastric cancer data. All cancer patients in the database were identified from Cancer Biomedical Informatics Grid (caBIG, http://cabig.cancer.gov/, microarray samples are published in the caArray project), the Gene Expression Omnibus (GEO, http://www.ncbi.nlm.nih.gov/geo/) and The Cancer Genome Atlas (TCGA, http://cancergenome.nih.gov) cancer datasets^19^. They collected clinical data including gender, perforation history, Lauren classification, differentiation, stage, HER2 status and treatment. The database was established using gene expression data and survival information of 876 gastric cancer patients downloaded from Gene Expression Omnibus (GEO). Briefly, four Notch sub-members (*Notch1, Notch2, Notch3 and Notch4)* were entered into the database (http://kmplot.com/analysis/index.php?p=service&cancer=gastric) to obtain Kaplan-Meier survival plots in which the number-at-risk is indicated below the main plot. Hazard ratio (HR) and 95% confidence intervals, as well as log rank P were calculated and displayed on the webpage. P value of <0.05 was considered to be statistically significant. HR is the ratio of the hazard rates corresponding to the conditions described by two levels of an explanatory variable in survival analysis.

## Results

**Figure 1:**
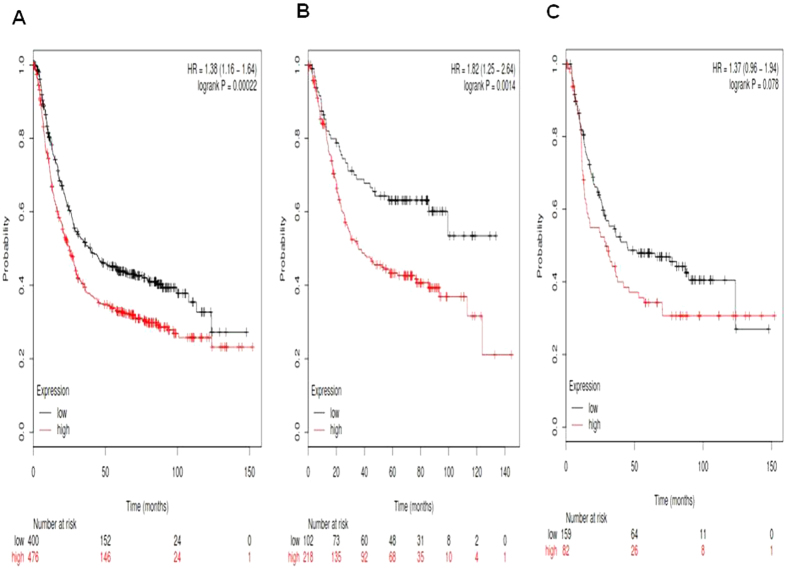
Determination of prognostic value of *Notch1* mRNA expression in www.kmplot.com. The desired Affymetrix IDs is valid: 218902_at (*Notch1*). (**A**) Survival curves are plotted for all gastric cancer patients (n = n = 876). (**B**) Survival curves are plotted for intestinal type cancer patients (n = 320). (**C**) Survival curves are plotted for diffuse type cancer patients (n = 241).

Mammals possess four different notch receptors, referred to as Notch1, Notch2, Notch3 and Notch4. All Notch receptors Kaplan-Meier survival information can be found in www.kmplot.com. We first accessed the prognostic value of *Notch1* mRNA expression in www.kmplot.com. The desired Affymetrix IDs is valid: 218902_at (*Notch1*). Survival curves were plotted for gastric cancer patients (n = 876) (Fig. 1A), for intestinal type cancer patients (n = 320) (Fig. 1B), and for diffuse type cancer patients (n = 241) (Fig. 1C). *Notch1*′*s* high mRNA expression was found to be correlated to worsen OS for all gastric cancer patients followed for 20 years, HR 1.38 (1.16–1.64), *p* = 0.00022. *Notch1* high mRNA expression was also found to be correlated to worsen OS in intestinal type cancer patients, HR 1.82 (1.25–2.64), *p* = 0.0014, but not in diffuse type cancer patients, HR 1.37 (0.96–1.94), *p* = 0.078.

**Figure 2:**
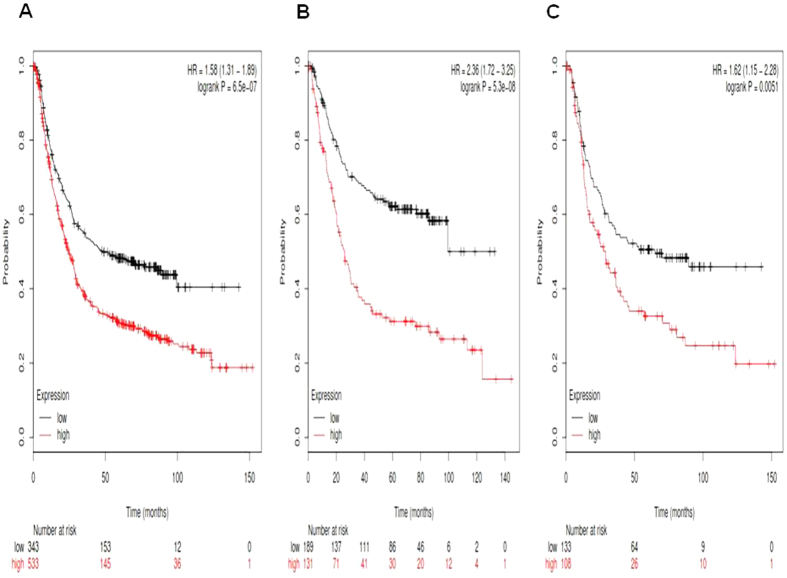
Determination of prognostic value of *Notch2* mRNA expression in www.kmplot.com. The desired Affymetrix IDs is valid: 210756_s_at (*Notch2*). (**A**) Survival curves are plotted for all gastric cancer patients (n = n = 876). (**B**) Survival curves are plotted for intestinal type cancer patients (n = 320). (**C**) Survival curves are plotted for diffuse type cancer patients (n = 241).

We then accessed the prognostic value of *Notch2* mRNA expression in www.kmplot.com. The desired Affymetrix IDs is valid: 210756_s_at (*Notch2*). *Notch2*′ high mRNA expression was found to be significantly correlated to worsen OS for all gastric cancer patients, HR 1.58 (1.31–1.89), *p* = 6.5e-07 (Fig. 2A), as well as in intestinal type cancer patients, HR 2.36 (1.72–3.25), *p* = 5.3e-08 (Fig. 2B), and in diffuse type cancer patients, HR 1.62 (1.15–2.28), *p* = 0.0051 (Fig. 2C).

**Figure 3:**
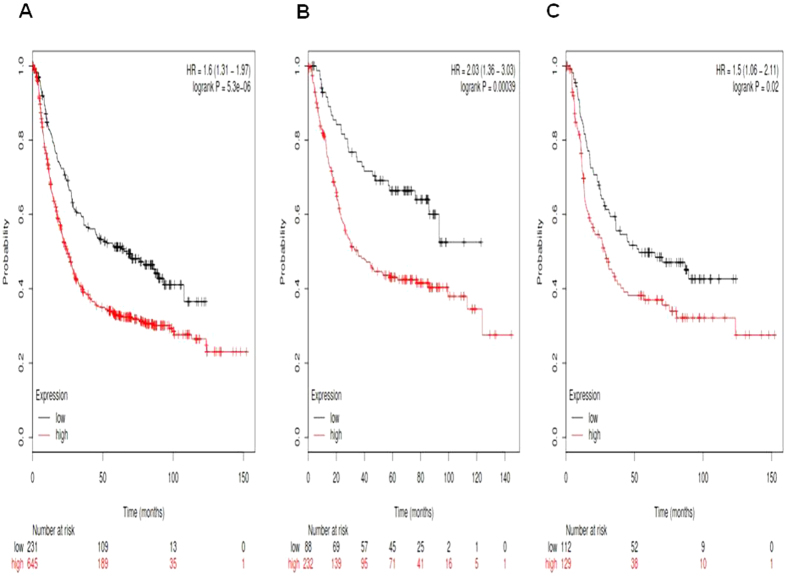
Determination of prognostic value of *Notch3* mRNA expression in www.kmplot.com. The desired Affymetrix IDs is valid: 203237_at (*Notch3*). (**A**) Survival curves are plotted for all gastric cancer patients (n = n = 876). (**B**) Survival curves are plotted for intestinal type cancer patients (n = 320). (**C**) Survival curves are plotted for diffuse type cancer patients (n = 241).

Figure 3 showed the prognostic value of *Notch3* mRNA expression in www.kmplot.com. The desired Affymetrix IDs is valid: 203237_at (*Notch3*). *Notch3*′ high mRNA expression was found to be significantly correlated to worsen OS for all gastric cancer patients, HR 1.6 (1.31–1.97), *p* = 5.3e-06 (Fig. 3A), as well as in intestinal type cancer patients, HR 2.03 (1.36–3.03), *p* = 0.00039 (Fig. 3B), and diffuse type cancer patients, HR 1.5 (1.06–2.11), *p* = 0.02 (Fig. 3C).

**Figure 4:**
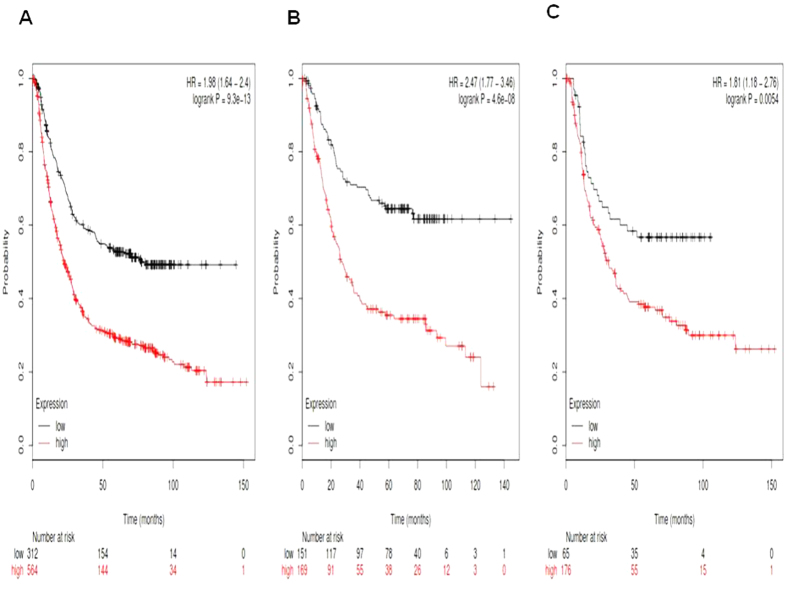
Determination of prognostic value of *Notch4* mRNA expression in www.kmplot.com. The desired Affymetrix IDs is valid: 205247_at (*Notch4*). (**A**) Survival curves are plotted for all gastric cancer patients (n = n = 876). (**B**) Survival curves are plotted for intestinal type cancer patients (n = 320). (**C**) Survival curves are plotted for diffuse type cancer patients (n = 241).

Figure 4 showed the prognostic value of *Notch4* mRNA expression in www.kmplot.com. The desired Affymetrix IDs is valid: 205247_at (*Notch4*). *Notch4*′*s* high mRNA expression was also found to be significantly correlated to worsen OS for all gastric cancer patients, HR 1.98 (1.64–2.4), *p* = 9.3e-13 (Fig. 4A), intestinal type cancer patients, HR 2.47 (1.77–3.64), *p* = 4.6e-08 (Fig. 4B), and diffuse type cancer patients, HR 1.81 (1.18–2.76), *p* = 0.0054 (Fig. 4C).

For further access to the correlation of individual Notch receptors with other clinicopathological features, we accessed the correlation with gender (Table 1), pathological grades (Table 2), clinical grades (Table 3), HER2 status (Table 4) and different choices of treatments (Table 5) of gastric cancer patients. As from Table 1, all the individual Notch receptors did not show significant difference of prognosis in different gender positive gastric cancer patients. From Table 2, all the individual Notch receptors except *Notch 2* were not significantly associated with pathological grades of gastric cancer patients. *Notch 2*′s high mRNA expression was associated with worsen OS in grade I gastric cancer patients, HR 10.5 (1.4–78.81), *p* = 0.0046. From Table 3, *Notch 1*′*s* high mRNA high expression was associated with worsen OS in grade II, HR 1.95 (1.04–3.64), *p* = 0.033 and grade III, HR 1.49 (1.07–2.06), *p* = 0.017. *Notch 2*′*s* high mRNA expression was associated with worsen OS in grade II, HR 2.26 (1.23–4.14), *p* = 0.0066 and grade III, HR 2.03 (1.46–2.82), *p* = 1.7e-05. *Notch 3*′*s* high mRNA expression was associated with worsen OS in grade III, HR 1.8 (1.26–2.57), *p* = 0.0011. *Notch 4*′*s* high mRNA expression was associated with worsen OS in grade I, HR 3.7 × 10^8^ (0.0-inf), *p* = 0.0035, grade II, HR 2.32 (1.26–4.25), *p* = 0.0052 and grade III, HR 1.83 (1.31–2.54), *p* = 0.00028. From Table 4, all the individual Notch receptors except *Notch 3* were significantly associated with worsen OS in either HER2 negative or HER2 positive gastric cancer patients. *Notch 3*′*s* high mRNA expression was only significantly associated with worsen OS in HER2 negative gastric cancer patients, HR 1.57 (1.22–2.01), *p* = 0.00043. From Table 5, *Notch 1*′*s* high mRNA expression was associated with worsen OS in surgery alone gastric cancer patients, HR 1.4 (1.02–1.93), *p* = 0.037; as well as 5-FU based adjuvant gastric cancer patients, HR 1.53 (1.07–2.19), *p* = 0.019. *Notch 2*′*s* high mRNA expression was only associated with better OS in 5-FU based adjuvant gastric cancer patients, HR 0.61 (0.43–0.87), *p* = 0.0059. *Notch 3*′*s* high mRNA expression was only associated with worsen OS in surgery alone gastric cancer patients, HR 1.42 (1.00–2.02), *p* = 0.048. *Notch 4* *s’* high mRNA expression was also only associated with worsen OS in surgery alone gastric cancer patients, HR 2.12 (1.48–3.03), *p* = 2.7e-05.

## Discussion

Among four Notch receptors and ligands, Notch1 is relatively the most studied member of Notch signaling in gastric carcinogenesis^7^,^37^,^38^,^39^. With the active form of Notch 1, the Notch 1 intracellular domain (NICD) was frequently expressed in gastric cancer cell lines, and the depletion of Notch 1 by Notch 1 siRNA led to growth inhibition of gastric cancer cells^17^,^40^. Down-regulation of Notch1 expression by gamma-secretase inhibition (N-[N-(3,5-difluorophenacetyl)-l-alanyl]-S-phenylglycine t-butyl ester, DAPT) was also able to substantially inhibit migration, invasion, and proliferation, as well as epithelial-mesenchymal transition in gastric cancer cell lines^41^. Changes in the expression of the Notch1 intracellular domain (NICD) differentially expressed in gastric cancer, and the aberrant expression of Notch1 NICD is associated with an advanced tumor stage, tumor metastasis and overall patient survival^42^. Du *et al.*^43^ performed a meta-analysis and showed that the expression of Notch1 protein was significantly higher in tumor tissues of gastric cancer compared to normal tissues. Specifically, stratified analyses showed that significantly increased expression of Notch1 was associated with non-cardia location, >5 cm size, diffuse type, positive lymphovascular invasion and distal metastasis, indicating that Notch1 protein may be an oncogene. Recently, Bauer *et al.*^44^ reported that primarily resected patients with Notch1 protein-negative tumors demonstrated worse survival and high Notch1protein expression was associated with early-stage tumors and associated with significantly increased survival in this subgroup. Their results supported that Notch1 expression indicated good prognosis in gastric cancer patients. However, whether or not *Notch1* mRNA has a prognostic role in gastric cancer patients remains elusive. In this report, *Notch1*′*s* high mRNA expression was found to be correlated to worsen OS for all gastric cancer patients followed for 20 years. *Notch1*′*s* high mRNA expression was also found to be correlated to worsen OS in intestinal type cancer patients, but not in diffuse type cancer patients.

Notch2 activation was observed in 10.0% (1 of 10) of noncancerous endoscopic mucosa, 71.4% (30 of 42) in premalignant lesions, and 97.3% (72 of 74) in gastric cancer tissues, demonstrating a correlation of Notch2 expression with both intestinal and diffuse gastric cancer formation^45^. Constitutive expression of Notch2 NICD promoted both cell proliferation and xenografted tumor growth of human gastric adenocarcinoma SC-M1 cells^46^. Immunohistochemical analysis demonstrated a chemotherapy-associated increase in the intensity of Notch2 staining, indicating a prominent role for Notch2 in chemotherapy resistance of gastric cancer^47^. Above results indicate that Notch2 seems to be a tumor oncogene in gastric carcinogenesis. Du *et al.*^43^ reported that the expression of Notch2 protein significantly was higher in tumor tissues of gastric cancer compared to normal tissues. However, Bauer *et al.*^44^ reported that higher Notch2 protein expression was associated with early-stage and intestinal-type tumors and with associated better survival in the subgroup of intestinal-type tumors. Their results support that Notch2 expression with early tumor stages suggest that Notch2 may act as a tumor suppressor in gastric cancer. However, there is no report about the prognostic significance of *Notch2* mRNA expression in gastric cancer. In this report, *Notch2*′*s* high mRNA expression was found to be significantly correlated to worsen OS for all gastric cancer patients, as well as in intestinal type cancer patients and in diffuse type cancer patients.

The study about Notch3 in gastric cancer is limited^43^. The expression of Notch3 protein was observed to be associated with the intestinal/glandular differentiation of gastric carcinoma cells, suggesting a role as a possible favorable prognostic indicator^48^. In this report, our results show that *Notch3*′*s* high mRNA expression was significantly correlated to worsen OS for all gastric cancer patients, as well as in intestinal type cancer patients and diffuse type cancer patients.

Same as Notch3, the study about Notch4 in gastric cancer is also limited. In a recent report^49^, Notch4 activation was observed to promote gastric cancer growth *in vitro* and *in vivo*, while Notch4 inhibition using Notch4 siRNA had opposite effects. In addition, Notch4 activation induced expression and activation of Wnt1, β-catenin and downstream target genes, c-Myc and cyclin D1, in gastric cancer cells, while Notch4 inhibition had opposite effects. Wnt1 is one of WNT members that regulates various processes including tumor initiation, tumor growth, cell senescence, cell death, differentiation and metastasis^50^,^51^. β-catenin is the primary transducer of canonical WNT signals to the nucleus and is involved in the development and progression of a significant proportion of gastric cancer cases^52^,^53^. Activation of the cyclin D1 oncogene, often by amplification or rearrangement, is a central mediator in the transition from G1 to S phase and a major driver of multiple types of human tumors including gastric cancer^54^,^55^,^56^. These results indicate that Notch4 seems to be a tumor oncogene in gastric carcinogenesis. In this report, we observed that *Notch4*′*s* high mRNA expression was also found to be significantly correlated to worsen OS for all gastric cancer patients, intestinal type cancer patients, and diffuse type of cancer patients.

The HER2*/neu* proto-oncogene (also known as *c-erbB-2*) encodes a 185 kDa transmembrane glycoprotein receptor known as HER2*/neu* or p185,^HER2^ partial homology with EGFR. HER2/*neu* shares a receptor with intrinsic tyrosine kinase activity and has been implicated in cancer with special emphasis in breast cancer^57^,^58^. HER2 overexpression was detected in 6% to 35% of GC patients and led to the advent of targeted therapy with anti HER2 antibody like Trastusumab which has improved the overall survival^59^,^60^. In this study, we found that all the individual Notch receptors except *Notch 3* are significantly associated with worsen OS in either HER2 negative or HER2 positive gastric cancer patients. *Notch 3*′*s* high mRNA expression is only significantly associated with worsen OS in HER2 negative gastric cancer patients.

The connection between Notch signaling and carcinogenesis, as well as its crosstalk with many oncogenic signaling pathways suggest that Notch signaling, especially some Notch receptors may be candidate for drug target of gastric cancer. A number of γ-secretase inhibitors were demonstrated to inhibit Notch activation and cell growth in gastric cancer cells^41^,^45^,^61^,^62^. The treatment combination of γ-secretase inhibitor and 5-FU was shown to be better than that with the single treatment in the inhibition of gastric cancer cell proliferation^61^. Therefore, Notch expression status could also impact the treatment efficiency of 5-FU based adjuvant therapy and/or prognosis of gastric cancer patients. In this study, we observed that *Notch 1* mRNA high expression is associated with worsen OS in surgery alone gastric cancer patients and 5-FU based adjuvant gastric cancer patients. *Notch 3* and *Notch 4*′*s* high mRNA expression is only associated with worsen OS in surgery alone for gastric cancer patients. Interestingly, *Notch 2*′*s* high mRNA expression is only associated with better OS in 5-FU based adjuvant gastric cancer patients.

In summary, by using the KM plotter database, we accessed the prognostic roles of four Notch receptors in gastric cancer patients through KM plotter, in which updated gene expression data and survival information included data from a total of 876 gastric cancer patients. All four Notch receptors’ high mRNA expression was found to be correlated to worsen overall survival (OS) for all gastric cancer patients followed for 20 years. We further accessed the prognostic roles of individual Notch receptors in different clinicopathological features including Lauren classification, pathological grades, clinical grades, HER2 status and different choices of treatments of gastric cancer patients. These results indicate that there are critical prognostic values of Notch 1–4 receptors in gastric cancer. This information will be useful for the better understanding of the heterogeneity and complexity in the molecular biology of gastric cancer and to develop tools to more accurately predict their prognosis.

## Additional Information

**How to cite this article**: Wu, X. *et al.* Prognostic values of four Notch receptor mRNA expression in gastric cancer. *Sci. Rep.* **6**, 28044; doi: 10.1038/srep28044 (2016).

## Figures and Tables

**Table 1:** Correlation of Notch receptor mRNA high expression with gender of gastric cancer patients.

Notch receptors	Treatment	Cases	HR 95% CI	P value
Notch1	Male	545	1.32 (1.06–1.63)	0.012
	Female	236	1.73 (1.22–2.47)	0.0021
Notch2	Male	545	1.9 (1.51–2.38)	1.8e-08
	Female	236	1.73 (1.12–2.67)	0.012
Notch3	Male	545	1.49 (1.16–1.92)	0.0019
	Female	236	1.71 (1.13–2.59)	0.011
Notch4	Male	545	2.21 (1.74–2.8)	3e-15
	Female	236	2.43 (1.47–4.01)	0.00032

**Table 2:** Correlation of Notch receptor mRNA high expression with pathological grades of gastric cancer patients.

Notch receptors	Pathological grades	Cases	HR 95% CI	P value
Notch 1	I	32	2.18 (0.9–5.28)	0.076
	II	67	0.76 (0.39–1.48)	0.41
	III	165	1.31 (0.84–2.05)	0.24
Notch 2	I	32	10.5 (1.4–78.81)	0.0046
	II	67	1.68 (0.87–3.21)	0.12
	III	165	1.37 (0.92–2.05)	0.12
Notch 3	I	32	1.28 (0.54–3.03)	0.57
	II	67	1.5 (0.76–2.97)	0.24
	III	165	1.3 (0.84–2.03)	0.24
Notch 4	I	32	1.72 (0.69–4.27)	0.24
	II	67	1.52 (0.79–2.94)	0.2
	III	165	0.8 (0.53–1.2)	0.28

**Table 3:** Correlation of Notch receptor mRNA high expression with clinical stages of gastric cancer patients.

Notch receptors	Clinical stages	Cases	HR 95% CI	P value
Notch1	1	67	3.17 (0.9–11.19)	0.058
	2	140	1.95 (1.04–3.64)	0.033
	3	305	1.49 (1.07–2.06)	0.017
	4	148	0.73 (0.49–1.07)	0.1
Notch2	1	67	2.1 (0.75–5.83)	0.15
	2	140	2.26 (1.23–4.14)	0.0066
	3	305	2.03 (1.46–2.82)	1.7e-05
	4	148	1.33 (0.87–2.03)	0.19
Notch3	1	67	2.76 (0.79–9.71)	0.098
	2	140	0.55 (0.28–1.08)	0.077
	3	305	1.8 (1.26–2.57)	0.0011
	4	148	1.4 (0.94–2.11)	0.1
Notch4	1	67	3.7 × 10^8^ (0.0-inf)	0.0035
	2	140	2.32 (1.26–4.25)	0.0052
	3	305	1.83 (1.31–2.54)	0.00028
	4	148	1.56 (0.97–2.51)	0.062

**Table 4:** Correlation of Notch receptor mRNA high expression with HER2 status of gastric cancer patients.

Notch receptors	HER2 status	Cases	HR 95% CI	P value
Notch1	Negative	532	1.32 (1.05–1.65)	0.016
	Positive	344	1.58 (1.2–2.09)	0.0011
Notch2	Negative	532	1.53 (1.22–1.93)	0.00023
	Positive	344	1.44 (1.07–1.96)	0.017
Notch3	Negative	532	1.57 (1.22–2.01)	0.00043
	Positive	344	1.31 (0.98–1.76)	0.065
Notch4	Negative	532	2.03 (1.61–2.57)	1.7e-09
	Positive	344	1.62 (1.25–2.1)	0.00025

**Table 5:** Correlation of Notch receptor mRNA high expression with different treatment of gastric cancer patients.

Notch receptors	Treatment	Cases	HR 95% CI	P value
Notch1	Surgery alone	380	1.4 (1.02–1.93)	0.037
	5-FU based Adjuvant	153	1.53 (1.07–2.19)	0.019
Notch2	Surgery alone	380	1.34 (0.98–1.85)	0.068
	5-FU based Adjuvant	153	0.61 (0.43–0.87)	0.0059
Notch3	Surgery alone	380	1.42 (1.00–2.02)	0.048
	5-FU based Adjuvant	153	0.82 (0.56–1.21)	0.31
	Surgery alone	380	2.12 (1.48–3.03)	2.7e-05
Notch4	5-FU based Adjuvant	153	1.22 (0.85–1.74)	0.27
